# Why studying intermodal duration discrimination matters

**DOI:** 10.3389/fpsyg.2014.00628

**Published:** 2014-07-01

**Authors:** Simon Grondin

**Affiliations:** École de psychologie, Université LavalQuébec, QC, Canada

**Keywords:** time perception, duration discrimination, psychological time, sensory modalities, attention

A critical issue in the field of time perception is whether or not explicit judgments about time are processed by some internal clock mechanism. A subsequent issue is whether or not this clock, if any, is central (i.e., is the same for a large range of durations, for whatever way of marking the intervals to be processed). There are several ways of marking time, including the use of signals delivered from different sensory modalities. In other words, do we have sensory specific representations of time, or is there an amodal—central—mechanism (Bueti, 2011)? This fundamental question is addressed here with an emphasis on the discrimination of brief empty time intervals. More specifically, intermodal intervals are of interest, an intermodal interval being marked by two brief and successive stimuli delivered from different sensory modalities.

The interest for the effect of modalities on perceived duration and sensitivity to time has grown recently. Researchers have reported that intervals marked by auditory signals are perceived as longer than time intervals marked by visual signals (Walker and Scott, 1981; Wearden et al., 1998; Penney et al., 2000; see Grondin, 2003), but this issue received recent attention in a context where auditory and visual signals marking time could be presented simultaneously (Gamache and Grondin, 2010; Hartcher-O'Brien et al., 2014). The relative duration of intermodal intervals also received attention. For instance, intervals marked by an audio-visual sequence are perceived as longer than intervals marked by a visuo-auditory sequence (Grondin and Rousseau, 1991; Grondin et al., 1996). Moreover, some intermodal experiments emphasized the role of markers' length on perceived duration (Grondin et al., 2005; Kuroda et al., 2014), with both the lengthening of the first and second marker resulting in longer perceived duration (Grondin et al., 1996).

Recently, Mayer et al. (2014) conducted an investigation involving intermodal intervals lasting from 100 to 900 ms, with combinations of auditory, visual and tactile (A, V, T) stimuli. They observed that when a sound serves as the first marker, in either an AV or AT sequence, duration is perceived as longer than in conditions where a sound serves as the second marker, as in a VA or TA sequence; but reported no ordering effect when tactile and visual signals were used together (TV vs. VT). Mayer et al. interpreted their results in terms of sensory latency (see also Grondin, 1993; Grondin et al., 1996), arguing that the summative distortion pattern they observed (by opposition to a multiplicative effect) is consistent with the hypothesis that there exists a central timekeeping mechanism, common for the processing of any intervals, independently of their markers' modality (see also Hartcher-O'Brien et al., 2014, for a similar conclusion). However, when intermodal and intramodal intervals are randomized from trial to trial, the overall interpretation in terms of sensory latency is more disputable (Grondin and Rousseau, 1991). For instance, for the discrimination of intervals circa 250 ms, Grondin and Rousseau (see their Table 6) reported a condition where the second marker of an interval was tactile, and the first was T, V, or A. They reported that an AT interval was perceived as much longer than TT and VT intervals. This could have been interpreted as if the A signal was detected more rapidly when serving as the first marker. However, when the second marker is always visual and the first one A, V, or T, it is not the AV intervals that are perceived as the longest, but the TV ones. In other words, an explanation based exclusively on latencies finds serious limitations when both intra- and intermodal intervals are compared.

Even more critical from a theoretical perspective is the question relative to the discrimination levels (sensitivity) reached with intermodal conditions. The recent data reported by Mayer et al. (2014) are also interesting as they describe the discrimination levels. In the VA and AV conditions, the Weber fractions are roughly the same, and vary from 30% at 0.1 s to slightly above 20% at 0.9 s. The results are essentially the same when auditory and tactile stimuli combinations are used, with the exception that performances are generally better when the auditory marker is presented first, especially at 0.1 s (above 40% in TA). With visual and tactile signals, the Weber fraction varies roughly between 25 and 31%, with the discrimination being usually better when the visual signal is presented first, especially at 0.1. For the discrimination of intervals lasting about 250 and 1000 ms, Rousseau et al. (1983) reported about the same performance levels in the VA and AV conditions. Also, for intervals lasting 1000 ms, Grondin (2003) reported about the same discrimination levels in AT and TA conditions, and in TV and VT conditions; at 250 ms, performance were slightly better in AT than in TA, and were slightly better in VT than in TV.

The stability of the Weber fraction over time reported by Mayer et al. (2014) is a bit surprising considering the data reported by Grondin (1996) for intermodal intervals lasting 0.125, 0.25, 0.5, 1, 2, or 4 s. In this study, AV and VA intervals were used. There were 36 sessions (3 per experimental condition) lasting about 30 (at 0.125 s) to 65 min (at 4 s). As reported in Figure 1, the performances in both conditions were roughly the same and, most importantly, the pattern over time was the same: there is an important and monotonic decrease of the WF from 0.1 s (much higher than 30%) to 1 s (circa 10%). Indeed, it is well-known that the WF is higher with briefer intervals, a fact that is accounted for by the generalized form of Weber's law (Grondin, 2001). In this experiment by Grondin (1996), explicit counting was not refrained, which should explain the low Weber fractions with longer intervals.

**Figure 1:**
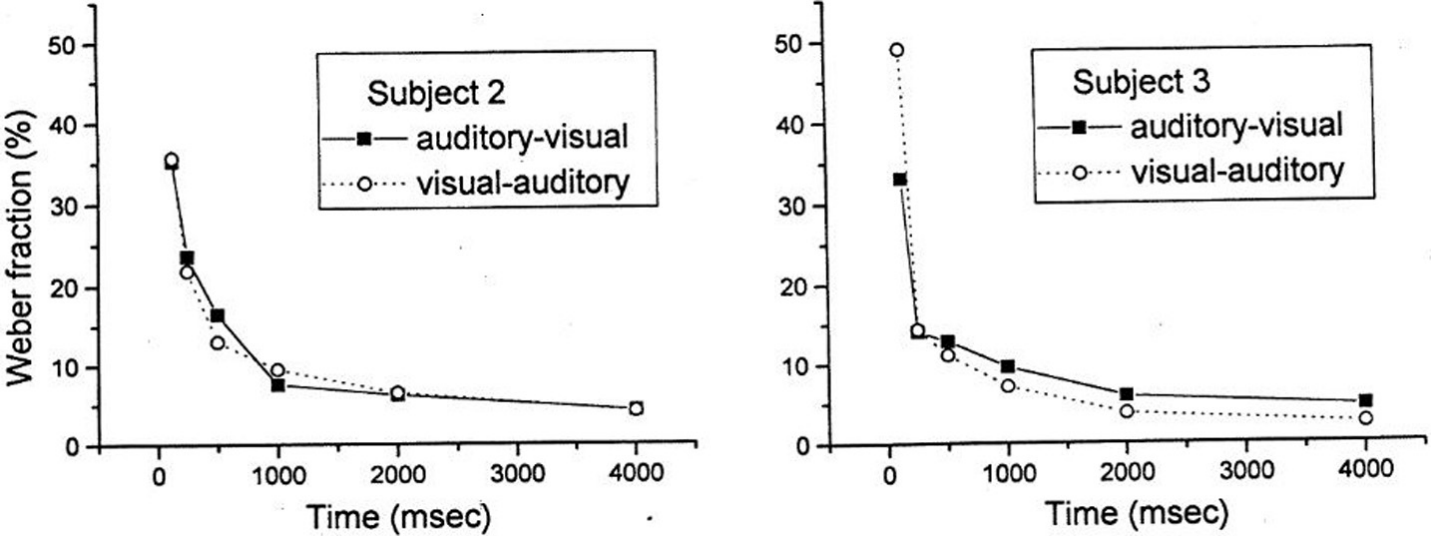
**Individual Weber functions for two intermodal conditions of duration discrimination (reported in Grondin, 1996)**.

Indeed, the real interest is not that much in the intermodal comparisons *per se*, but in the comparison of intra- and intermodal intervals. It is well-established that the discrimination of time intervals is much easier with auditory than with visual markers (Grondin et al., 2001, 2008; Grondin, 2005). If this auditory vs. visual difference is due to the sensory noise associated with the signals marking an interval, marking an empty interval with one auditory signal and one visual signal (AV or VA) should lead to performance levels in-between the ones involving two auditory (AA) and two visual (VV) signals. In Grondin (1993), the Weber fraction for the same range of durations tended to increase with briefer intervals (0.125 s)—especially when using visual signals—varying between 4 and 8% in AA, and circa 12% in VV. An interpretation in terms of variability (or latencies) belonging to the signals themselves would predict a performance level (WF) between 8 and 12%. This result is far from the Weber fraction above 30% reported by Grondin (1996) for intermodal intervals with the same method.

This intra- vs. intermodal difference challenges another hypothesis. Using transcranial magnetic stimulations (TMS) over the primary auditory cortex, Kanai et al. (2011) observed that time discrimination is impaired not only when auditory signals mark time, but also when visual signals do. However, only the performance in the visual condition is impaired when TMS is used over the primary visual cortex. This finding suggests that in timing tasks, the auditory cortex has a supramodal role: the lower performance level in vision than in audition would be due to the need to transfer the visual signals into an auditory code (Kanai et al., 2011). If such is the case though, having one auditory signal (AV or VA conditions) instead of none (VV) should lead once again to performance levels in-between the ones involving two auditory and two visual signals. However, clearly, for very brief intervals (<1 s) and when A and V signals are used, discrimination is severely impaired in AV and VA conditions compared with AA and VV intramodal conditions (Rousseau et al., 1983; Grondin and Rousseau, 1991; Grondin et al., 2005).

Note however that for intervals lasting 1.6 s, the large difference between the threshold value in AA and AV conditions is washed out when an explicit count of numbers is used for completing the task (Grondin et al., 2004). This reduction could certainly be attributed to the efficiency of using sub-intervals (smaller chunks of information), assuming that the counting process remains error free. However, the hypothesis that the efficiency of counting is actually due to the translation of visual signals into an auditory code cannot be discarded.

Recent EEG data, and more specifically the amplitude of the contingent negative variations recorded at fronto-central electrodes, revealed a basic difference between the AA condition and other modality conditions (Gontier et al., 2013; Hasuo et al., 2014). There seems to be something specific to auditory time perception. Moreover, an attentional component would also be at the heart of the intra- vs. intermodality differences (Gontier et al., 2013).

In brief, the different perceived durations and discrimination levels observed in the different intra- and intermodal conditions is a challenge for the single-clock hypothesis. An interpretation based only on sensory latencies (Mayer et al., 2014) would not be sufficient to account for the intra- vs. intermodal difference. Indeed, it would be difficult to explain the variance observed in all intra- and intermodal conditions, for both perceived duration and discrimination levels (Grondin, 1998), on the basis of the variance due to the clock process, and to the addition of non-temporal noise (sensory latencies or attention switching). However, before concluding that there is some modality-specific temporal processing instead of a central clock, it remains necessary to understand the real impact of all the possible interactions amongst the sources of non-temporal noise (how a stimulus in one modality impacts the detection of the attention to a stimulus to be delivered in other modalities, what is the role of prior entry in intramodal conditions, …). Another avenue is the possibility to have hierarchical model involving a level with modality-specific temporal processing and modality-independent processing system at another level (Stauffer et al., 2012).

## Conflict of interest statement

The author declares that the research was conducted in the absence of any commercial or financial relationships that could be construed as a potential conflict of interest.
